# Group foraging in Socotra cormorants: A biologging approach to the study of a complex behavior

**DOI:** 10.1002/ece3.2750

**Published:** 2017-02-26

**Authors:** Timothée R. Cook, Rob Gubiani, Peter G. Ryan, Sabir B. Muzaffar

**Affiliations:** ^1^ Department of Evolutionary EcologyEvolutionary Ecophysiology TeamInstitute of Ecology and Environmental SciencesUniversity Pierre et Marie CurieParisFrance; ^2^ FitzPatrick Institute of African OrnithologyDST‐NRF Centre of ExcellenceUniversity of Cape TownRondeboschSouth Africa; ^3^ Department of BiologyUnited Arab Emirates UniversityAl AinUnited Arab Emirates

**Keywords:** anchovy, communal, cooperative, fisheries, sardine, social

## Abstract

Group foraging contradicts classic ecological theory because intraspecific competition normally increases with aggregation. Hence, there should be evolutionary benefits to group foraging. The study of group foraging in the field remains challenging however, because of the large number of individuals involved and the remoteness of the interactions to the observer. Biologging represents a cost‐effective solution to these methodological issues. By deploying GPS and temperature–depth loggers on individuals over a period of several consecutive days, we investigated intraspecific foraging interactions in the Socotra cormorant *Phalacrocorax nigrogularis*, a threatened colonial seabird endemic to the Arabian Peninsula. In particular, we examined how closely birds from the same colony associated with each other spatially when they were at sea at the same time and the distance between foraging dives at different periods of the day. Results show that the position of different birds overlapped substantially, all birds targeting the same general foraging grounds throughout the day, likely following the same school of fish. There were as many as 44,500 birds within the foraging flock at sea at any time (50% of the colony), and flocking density was high, with distance between birds ranging from 8 to 1,380 m. Birds adopted a diving strategy maximizing time spent underwater relative to surface time, resulting in up to 72% of birds underwater in potential contact with prey at all times while foraging. Our data suggest that the benefits of group foraging outweigh the costs of intense aggregation in this seabird. Prey detection and information transmission are facilitated in large groups. Once discovered, shoaling prey are concentrated under the effect of the multitude. Fish school cohesiveness is then disorganized by continuous attacks of diving birds to facilitate prey capture. Decreasing population size could pose a risk to the persistence of threatened seabirds where group size is important for foraging success.

## 1 Introduction

Many predator species aggregate at the intraspecific level for the purpose of foraging, a phenomenon known variously as communal foraging, cooperative foraging, social foraging, or more generally as group foraging. For group foraging to be an evolutionarily advantageous strategy, its benefits should outweigh the costs of increased competition for food resulting from aggregation (for a review, see Beauchamp, 2014). Despite its obvious relevance to different fields of ecology, the study of group foraging in predators remains challenging. This is due to the study model itself, which may comprise anything from half a dozen to billions of individuals interacting closely in space and time (e.g., Creel & Creel, 1995; Duffy, 1983; Radakov, 1973). Furthermore, foraging groups tend to target prey that aggregate in vast numbers. Thus, following the behavior of each animal individually within the flock, herd or school would seem virtually impossible. Additionally, groups form and feed in areas usually inaccessible to the human eye. Consequently, most studies have used a modeling approach (e.g., Bhattacharya & Vicsek, 2014; Giraldeau & Caraco, 2000; van der Post & Semmann, 2011; Silk, Croft, Tregenza, & Bearhop, 2014), or to a lesser extent an experimental approach (Bijleveld, van Gils, Jouta, & Piersma, 2015; Carthey & Banks, 2015; Ekman & Hake, 1988; Fernández‐Juricic, Siller, & Kacelnik, 2004; Saino, 1994), but there are few field studies (Brown, 1988; Creel & Creel, 1995; MacNulty, Smith, Mech, Vucetich, & Packer, 2012).

Seabirds are an interesting group for the study of group foraging because, in many species, individuals aggregate year round, whether on land (breeding and roosting) or at sea (rafting and foraging) (Schreiber & Burger, 2002). Hence, in such species, important benefits are expected to compensate the costs of coloniality (e.g., competition for partners or breeding space, and transmission of disease) and of foraging (intraspecific competition for food). Benefits are thought to include increased knowledge about location and quality of foraging grounds, via public information and local enhancement (e.g., Buckley, 1997; Danchin & Wagner 1997; Bairos‐Novak, Crook, & Davoren, 2015), and increased success of prey capture, via depolarization of fish schools under the combined attack of many individuals (Wilson, Ryan, James, & Wilson, 1987). Group‐foraging seabird species, such as the Guanay cormorant (*Phalacrocorax bougainvillii*, Weimerskirch, Bertrand, Silva, Bost, & Peraltilla, 2012) or the Cape cormorant (*Phalacrocorax capensis*, Cook et al., 2012), tend to specialize on shoaling prey, usually small epipelagic fish, but also on krill, as in the case of the short‐tailed shearwater (*Puffinus tenuirostris*, Hunt, Coyle, Hoffman, Decker, & Flint, 1996) or murres (*Uria* spp., Hunt, Harrison, Hamner, & Obst, 1988). These seabirds may aggregate into foraging flocks of up to several hundred thousand individuals (Gould, Forsell, & Lensink, 1982), suggesting they rely on extremely high prey densities. This dependency on an important and highly concentrated biomass of “forage fish” is considered one of the causes of the decline of several bird species, due to competition with pelagic fisheries (Hobday, Bell, Cook, Gasalla, & Weng, 2015). A better understanding of the processes underlying group foraging in seabirds is thus needed.

Studying group foraging in seabirds is typically complex, not just because of the number of individuals involved, but also because individuals typically forage out of human sight, that is, out to sea and often underwater. Until recently, observations of seabird aggregations were rendered possible primarily with the use of aerial‐based (e.g., Buckland et al., 2012; Certain & Bretagnolle, 2008) or vessel‐based (e.g., Duffy, 1989; Ronconi & Burger, 2009) surveys. Such studies are essential in understanding which species aggregate and where, and in flock size estimation. However, they only give a snapshot of the behavioral processes under way at the time of the survey (but see Piatt, 1990). They also may introduce an observational bias as some species may be attracted by the vessel, depending on its type and activity (Bodey et al., 2014). Underwater filming can provide detailed understanding of the interactions between a flock and a bait ball (Thiebault, Semeria, Lett, & Tremblay, 2016). Yet, the logistical constraints and observational biases inherent in this method limit its more general use. Radar has the potential to follow flocks and even individual birds over an extended time frame; however, species identification may be uncertain, and surveillance range is limited to ca 10 km (Gauthreaux & Belser, 2003).

Recently, animal‐attached remote sensing, otherwise known as “biologging” (Ropert‐Coudert & Wilson, 2005), has enabled the description of fine‐scale behavior in free‐ranging animals. Although biologging cannot replace at‐sea surveys for studying some of the behavior of seabirds at foraging grounds or for estimating the size of aggregations, it provides high‐resolution behavioral observations and enables following birds individually, over several days, something which surveys cannot do. Miniaturized electronic devices recording behavior have already been used to study group foraging in some seabird species. Time–depth recorders have shown that penguins feeding on pelagic prey sometimes dive synchronously (Berlincourt & Arnould, 2014; Takahashi, Sato, Nishikawa, Watanuki, & Naito, 2004; Tremblay & Cherel, 1999), thus confirming observations that penguins may forage in flocks (Wilson, Wilson, & McQuaid, 1986) and simultaneously revealing a coordinated underwater foraging behavior based on constant visual contact between birds. Acting as a unit, birds are capable of depolarizing the fish school, thus presumably reducing its coordinated antipredator behavior and potentially facilitating prey capture (Wilson et al., 1987). Miniaturized cameras mounted on pursuit‐diving species have allowed further investigation of the relationship between predator foraging success and predator group size (Takahashi, Sato, Naito, et al., 2004). Some penguins may experience an equivalent or higher foraging success when foraging alone on a fish school than when foraging as a group on the same resource (Sutton, Hoskins & Arnould 2015). Therefore, in some cases, the benefits of group foraging may derive more from an increased probability of detecting prey by associating with conspecifics than from an increased prey capture rate. GPS tags deployed on penguins have shown that the duration of association between individuals is highly variable (Berlincourt & Arnould, 2014). Some birds leave the colony together and spend the entire foraging trip in close association, whereas others only meet up briefly at foraging patches, with birds already at sea possibly attracting new birds to active foraging areas. Publicly relayed information regarding the location of food patches is presumably more important in flighted species, however, because the probability of detecting another bird increases with altitude. Cameras mounted on Cape gannets (*Morus capensis*) have shown how the presence of other seabirds nearby causes birds to change course during flight, decreasing the time to first prey encounter (Thiebault, Mullers, Pistorius, & Tremblay, 2014; Thiebault & Tremblay, 2013; Thiebault, Mullers, Pistorius, Meza‐Torres, et al., 2014; Tremblay, Thiebault, Mullers, & Pistorius, 2014). Hence, on a small scale, individuals may not be looking for the prey itself as much as using local enhancement as a mechanism guiding their movement patterns.

Biologging has thus proven to be an efficient (and cost‐effective) tool for studying group foraging in penguins and gannets. However, more biologging studies are needed for a more general understanding of group foraging across different seabird taxa. Cormorants, for example, are a family represented by several species that rely on group foraging (Nelson, 2006; Orta, 1992). We studied the vulnerable Socotra cormorant (*Phalacrocorax nigrogularis*), a species endemic to the Arabian Gulf known for large at‐sea aggregations (Jennings, 2010), but for which there is little information on its foraging behavior. We used a biologging approach to study group foraging in this species by deploying GPS and temperature–depth recorders on breeding adults, thus accurately measuring their behavior in three dimensions and with detailed temporal resolution.

## 2 Materials and Methods

### 2.1 Study area

**Figure 1:**
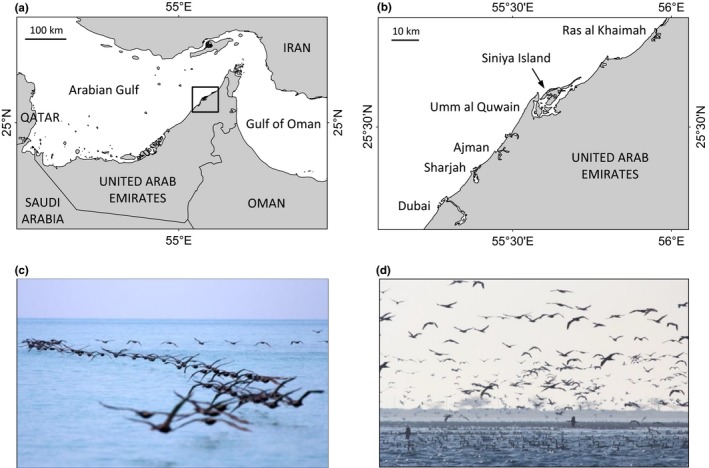
Location of the study colony and at‐sea aggregations of Socotra cormorants. (a) Map of the Arabian (Persian) Gulf and Gulf of Oman area. (b) Close‐up of the northeast of the United Arab Emirates showing the position of Siniya Island hosting the study colony. (c) Cormorants commute to and from foraging grounds in groups comprising hundreds of individual streams of 10–50 birds flying in tight formation. (d) At foraging grounds, cormorants aggregate at the sea surface by the thousands. Photographs: Rob Gubiani

Fieldwork was performed on Siniya Island (25°37′N, 55°37′E; Figure 1a,b). Siniya is an island that is part of a more extensive system of islands and lagoons dominated by mangroves (*Avicennia marina*) and hosting other emblematic bird species, such as the greater flamingo (*Phoenicopterus roseus*). Although it is a major breeding locality for the Socotra cormorant, an important refuge for other terrestrial and marine wildlife and one of the last undisturbed coastal ecosystems of the Emirates, Siniya, and its attending habitat is threatened by human development (Sheppard et al., 2010).

### 2.2 Study model

The Socotra cormorant is an average‐sized flighted pursuit‐diving seabird endemic to the Arabian Gulf, the Gulf of Oman, and the southeast coast of the Arabian Peninsula. It is declining and classified as vulnerable (BirdLife International 2012). About 33,000 pairs breed annually from September–December on Siniya, making it one of the three most important breeding localities for this seabird (Muzaffar, unpublished data). Little is known about its foraging ecology. At‐sea observations have described large aggregations of individuals foraging communally (Jennings, 2010; Figure 1c,d). Dietary analyses at the colony have shown that Socotra cormorants forage on shoaling fish, mainly anchovy (*Encrasicholina* spp.), bluestripe herring (*Herklotsichthys quadrimaculatus*), and African sailfin flying fish (*Parexocoetus mento*), suggesting little overlap with local fisheries (Muzaffar et al., 2016).

### 2.3 Deployment of data loggers

We studied adult Socotra cormorants breeding on Siniya between 11 and 26 November 2012, and 10 and 19 November 2013. Within the colony, study nests were chosen as far from one another as possible in order to avoid multiple disturbances to any one study bird. Birds were captured by the feet on their nest using nooses triggered from a distance. Each noose was set around the edge of the nest, which is a cup built in sand. Birds were captured during the late incubation/hatchling stage because eggs and hatchlings (naked altricial chicks younger than 2 weeks old, following Gubiani, Benjamin, & Muzaffar, 2012) are not in danger of being caught in the noose, while older chicks may accidently become snared because of their larger size. Culmen and tarsus of adults were measured using a Vernier caliper. Adult wing length (flattened chord) was measured with a stopped ruler. Adults were weighed inside a bag using a spring balance to the nearest 10 g: average adult body mass was 1.52 ± 0.15 kg (range: 1.30–1.82 kg). Study birds were 70% females and 30% males (Table S1).

GPS loggers and temperature–depth recorders (TDRs) were deployed simultaneously on a total of 20 birds (11 in 2012 and nine in 2013). The GPS loggers were attached to coverts of the lower back using Tesa tape No. 4651 (Beiersdorf AG, Hamburg) following Wilson et al. (1997), while TDRs were attached under the three central rectrices with Tesa tape. GPS loggers were CatTrack 1 models (Catnip Technologies Ltd, Hong Kong) encased in a heat shrink tube. They weighed 19 g, measured 1 × 2.5 × 4.5 cm, and recorded position with an accuracy of ~10 m in field conditions. TDRs were Cefas G5 models (Cefas Technology Ltd., Lowestoft). They weighed 2.7 g, measured 0.8 × 3.1 cm, and recorded temperature and depth with an accuracy of 0.1°C and 0.5 m. Combined weight of both loggers represented 1.4 ± 0.1% (range: 1.2–1.7%) of the study birds' body mass, which is below the threshold of 2–3% recommended for flying birds (Phillips, Xavier, & Croxall, 2003). In order to optimize the trade‐off between recording duration, battery life, and data resolution, GPS loggers were programmed to sample every 20 s and to stop recording at night when birds were at the colony and inactive. TDRs were programmed to continuously record depth and temperature at an interval of 1 s. Birds were recaptured and data loggers retrieved after 2–5 days, depending on the timing of visits to island and duration of GPS batteries. All procedures complied with the guidelines of the Ministry of Environment and Water and the United Arab Emirates University's Animal Ethics Committee.

### 2.4 Analysis of tracking data

GPS data were analyzed in ArcGIS 10.2 for desktop (Esri^®^ ArcMap^™^ 10.2.0). A foraging trip was defined as beginning when a bird left the colony for the sea and ending when it returned to the colony. Maximum foraging distance from the colony was calculated as the maximum distance in a straight line reached during a trip. Path length was measured as the sum of distances between all successive GPS points during a trip. Ground speed was calculated as the instantaneous speed between two successive GPS points. Based on the frequency distribution of ground speeds, we established a cutoff value at 15 km/hr to discriminate between flying and nonflying behavior (Figure S1). It was not possible to detect short flights because of the sampling rate of the GPS. Therefore, short flights were determined in MultiTrace (Jensen Software Systems, Laboe, Germany) using the temperature data recorded by TDRs. Following Tremblay, Cherel, Oremus, Tveraa, and Chastel (2003) and Tremblay, Cook, and Cherel (2005), these flights were detected to the nearest second based on the temperature contrast between air and water. Flight onset was associated with the windchill effect on the wet temperature sensor when birds left the water surface, and flight end was determined by the sudden temperature increase when birds alighted on the water surface (Figure S2). This was confirmed by similar temperature variations recorded in long flights detected by GPS.

Timing of dives was used to determine dive coordinates from tracking data. Due to the difference between GPS and TDR sampling intervals and loss of positional information when birds were submerged, dives were assigned the closest recorded GPS position, but only if this position was recorded ≤2 min from when the dive occurred, a threshold short enough to exclude significant movements of birds (Cook et al., 2012). An analysis of the density distribution of dives was conducted in R 3.0.3 (R Core Team 2014) using ks, the kernel smoothing package (Duong, 2007) which implements diagonal and unconstrained data‐driven bandwidth matrices (smoothing parameters) for kernel density estimation.

### 2.5 Analysis of foraging area consistency

One way to explore the extent to which birds forage consistently in the same area is to calculate the distance between foraging areas of birds that are known to have foraging trips which overlap in time. Because average foraging trip duration was ca 4 hr (Figure S3), birds that left the colony ≥4 hr apart were less likely to have foraging trips which overlapped in time. Furthermore, frequency distribution of time of departure from the colony was trimodal, with a peak at 8, 12, and 15 hr (Figure S3). We therefore divided days into three 4‐hr periods (6–10 hr, 10–14 hr, and 14–18 hr) and then calculated, for each trip, the distance in foraging area location (computed as the barycenter of the positions of all foraging dives recorded during the trip) between all birds that left to sea during the same period. In order to explore consistency in foraging area over time, we also calculated the distance between foraging locations of birds for foraging trips on the same day and from 1 day to the next.

### 2.6 Analysis of foraging interactions

Further detailed analysis of group foraging was carried out by studying interactions between individuals at sea. For this, we measured how closely birds interacted in a subset of eight bird “pairs” (12 individuals) when the foraging path of both pair members was almost perfectly superimposed. For each pair, we calculated the distance between birds at any given time, but only when both pair members were at sea. A bird could join its matching pair member (assumed to be in a flock) after the latter had already been foraging for several minutes or hours. We therefore focused on the distance between birds when they were presumed to be interacting within the group. We called this distance intraflock association distance (IFAD). Calculation of IFAD started arbitrarily when birds were within 1 km of each other for the first time and ended when they were within 1 km of each other for the last time.

### 2.7 Analysis of dive data

Time–depth data were analyzed in MultiTrace‐Dive (Jensen Software Systems, Laboe, Germany). After correcting for the drift of the depth sensor (zero‐offset correction), dives were considered to occur when depth was ≥0.2 m. Dive duration was calculated between the start and the end of the dive. Maximum dive depth was the maximum depth reached by the bird during the dive. Postdive interval (surface recovery phase) was defined as the time between the end of the dive and the start of the next dive, unless a flight was detected in between, in which case the postdive interval ended when the flight began (Tremblay et al., 2005). Intervals >100 s (3.2%) were not considered to represent postdive surface recovery/predive preparation events, but other surface activities (Tremblay et al., 2005) due to a break in the distribution of postdive intervals (Figure S4). Descent and ascent phases took place between the start of the dive and the start of the bottom phase and between the end of the bottom phase and the end of the dive, respectively. The start and end of the bottom phase were determined when vertical transit rates were ≤0.25 and ≥0.25 m/s, respectively (Kato, Ropert‐Coudert, Grémillet, & Cannell, 2006). In order to account for the effect of windchill on the temperature sensor during flights preceding dives, water temperature was defined as maximum water temperature recorded during a dive.

Dive shape was determined visually in MultiTrace‐Dive. Because dive shape depends on the scale at which the dive profile is being observed, dive shapes were determined at all times with the graph window showing 10 min on timescale and 20 m on vertical scale (or 10 m when dives were <10 m) (Cook et al., 2012). Dive shapes were placed into four categories: flat‐bottomed dives, V‐shaped dives, parabola‐shaped dives, and irregular dives (Cook et al., 2012; Wilson, Culik, Peters, & Bannasch, 1996). Time–depth profiles of birds that were at sea together were graphically superimposed to assess whether they dived synchronously. Synchronous diving has been detected in small groups of diving penguins and is believed to increase foraging efficiency of birds feeding off shoaling prey (Berlincourt & Arnould, 2014; Takahashi, Sato, Nishikawa, et al., 2004; Tremblay & Cherel, 1999).

### 2.8 Statistics

We performed linear mixed‐effects model in R using the “nlme” package. We first tested the effect of year on foraging parameters, setting bird as a random effect. Year was first set as a covariate, but was dropped from the final model as it had no effect. Different foraging parameters tested included time of trip start, time of trip end, trip duration, maximum linear distance to colony, path length, bearing, time flying per trip, time at the sea surface per trip, time diving per trip, number of dives per trip, maximum dive depth, dive duration, postdive interval, and maximum water temperature during dive. Because gender can have an effect on activity rhythms in some cormorant species (Cook, Lescroël, Cherel, Kato, & Bost, 2013), we also tested the effect of sex on time of departure from the colony. Using the same model framework, we then tested the effect of trip phase (outbound or inbound) on four variables (log‐transformed): phase duration, proportion of phase time spent on and under the water surface, flight speed, and average bird velocity (linear distance between the starting and ending points of the phase/phase duration). Eventually, we tested the effect of time (trip order and day) on the distance (log‐transformed) between the barycenter of foraging dive locations on successive trips.

Data are graphically presented as mean ± standard deviation per data interval (bin), but all regressions were fitted on the raw data. In the text, results are reported as mean ± standard deviation.

## 3 Results

### 3.1 General foraging behavior

The 20 study birds completed 50 foraging trips and 5,225 dives over the two study periods. Foraging trip parameters are summarized in Table 1. Detailed individual foraging parameters are presented in Tables S2–S4.

**Table 1:** Summary of foraging trip parameters of Socotra cormorants breeding on Siniya Island (*n* = 50 trips)

	Mean	*SD*	Min.	Max.
Individual trips per day	1.1	0.3	1	2
Dives per trip	104.5	52.7	1	240
Foraging flights^a^ per trip	27	13	1	68
Daily time diving (hr)	0.8	0.3	0.20	1.7
Daily time on sea surface (hr)	1.7	0.8	0.6	3.6
Daily time flying (hr)	1.9	0.9	0.3	4.6
Trip departure time (hr:min)	11:05	02:38	06:22	16:27
Trip return time (hr:min)	14:58	02:29	09:25	18:25
Trip duration (hr)	3.7	1.5	0.9	7.4
Max. distance to colony (km)	32.6	20.2	2.8	63.9
Foraging path length (km)	80	45.3	7.5	157.1
Flight speed (km/hr)	45.2	10.6	15	89.2

a Flights occurring between the first and the last dive of a trip.

**Figure 2:**
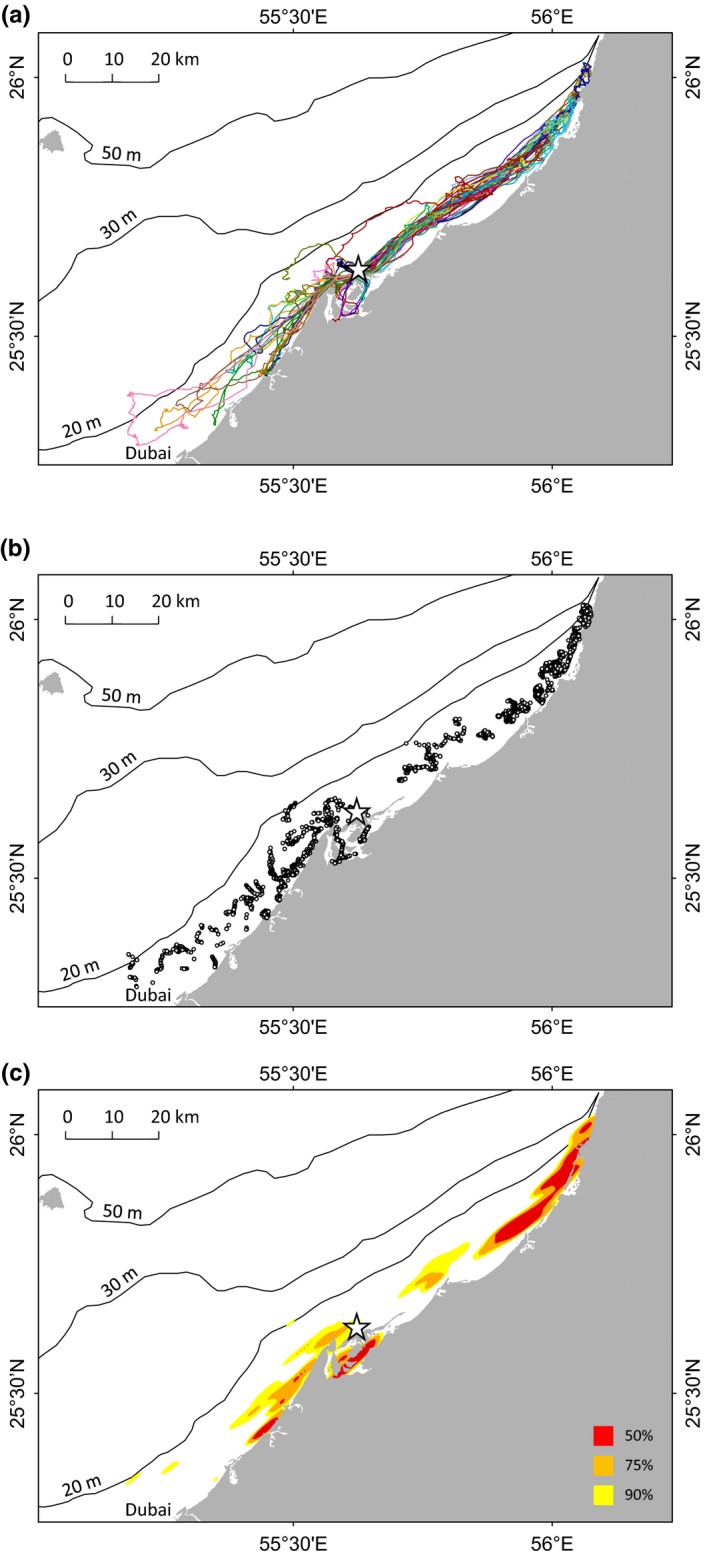
Foraging areas of Socotra cormorants (*n* = 20) breeding at Siniya Island (white star) in 2012 and 2013. (a) Foraging tracks (*n* = 50), (b) dives (*n* = 4,966), and (c) dive distribution kernel density plots

Birds foraged up and down the coastline, following two main bearings of 45° and 235° (Figure S5), never venturing farther than 18 km offshore (Figure 2a). Geographic coordinates were determined for 95% of dives. Birds dived almost exclusively over seafloor depths ≤20 m (Figure 2b). Kernel density plots show that most dives occurred in waters ≤10 m deep and that birds concentrated their foraging effort just southwest of the colony, between Siniya Island and Ajman, and 40–50 km to the northeast, around Ras al Khaimah (Figure 2c). Year had no effect on foraging parameters (Table S5). Sex had no effect on time of bird departure from the colony (*df* = 18, *t* = 0.02, *P* = .982), with 63% of males and 57% of females departing before noon.

**Figure 3:**
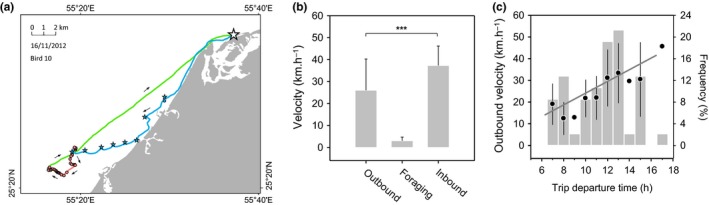
Different phases of Socotra cormorant foraging trips and associated bird velocity. (a) Structure of a typical foraging trip, including the outbound phase (blue), the foraging phase (pink), and the inbound phase (green). The white star corresponds to Siniya colony. Stars along the outbound path mark places where birds landed on the water surface (no stopover during the inbound phase). Circles correspond to dives; in this example, the bird carried out one prospective dive during the outbound phase and 52 dives during the foraging phase. (b) Average bird velocity (linear distance between the starting and ending points of the phase/phase duration) for the different trip phases (***P < .001). (c) Average bird velocity during the outbound phase as a function of trip departure time (*y *=* *2.7*x* − 3.43, *R* ^2^ = .22, *P* < .0001, *n* = 47) and frequency distribution of trip departure time (gray vertical bars, *n* = 47)

Foraging trips were typically composed of an outbound commuting phase from the colony to the foraging ground, a foraging phase with intense diving activity and an inbound commuting phase from the last foraging dive back to the colony (Figure 3a). The outbound phase lasted longer (1.3 ± 0.7 hr) than the inbound phase (0.9 ± 0.5 hr) (*df* = 73, *t* = 2.11, *P* = .038), while the foraging phase lasted 1.7 ± 1 hr on average. Birds landed more often on the sea surface during the outbound (4.1 ± 4.8 landings per trip) than during the inbound (1.6 ± 4.9 landings per trip) phase. They also dived occasionally during the outbound phase (but not during the inbound phase): such dives were considered to be prospective dives, as opposed to the foraging dives characterizing the foraging phase (Figure 3a). Prospective dives were present in 49% of trips (6 ± 10 dives per trip), amounting to 0.05% of all dives carried out by birds (foraging dives added to 97 ± 50 dives per trip). Birds spent more time at the sea surface and underwater during the outbound phase (42.3 ± 25.9%) than during the inbound phase (17.8 ± 17.2%) (*df* = 73, *t* = 5.99, *P* < .0001), a proportion that was 82.8 ± 10.8% during the foraging phase. Bird instantaneous flight speed was significantly slower during the outbound phase (45.4 ± 7.1 km/hr) than during the inbound phase (48.6 ± 6.1 km/hr) (*df* = 73, *t* = −2.4, *P* = .018), while it was an average of 34.7 ± 6.0 km/hr during the foraging phase. As a result of these differences, bird velocity was 11 km/hr slower on average during the outbound than during the inbound phase (*df* = 73, *t* = −4.92, *P* < .001) (Figure 3b). Bird velocity during the outbound phase increased linearly with trip departure time (Figure 3c).

### 3.2 Foraging area consistency

Foraging behavior of individual birds was recorded over 1–4 consecutive days. Most study birds carried out trips on days when other study birds were also at sea: 90%, 56%, 48%, 38%, and 26% of foraging trips occurred on days when at least 2, 3, 4, 5, and 6 birds were at sea, respectively.

**Figure 4:**
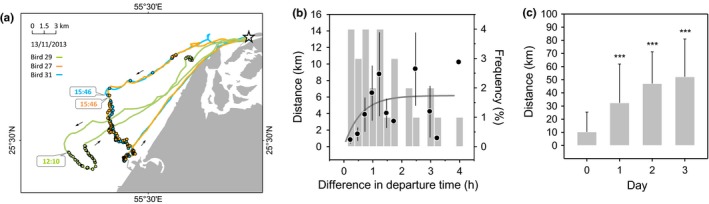
Foraging area consistency in Socotra cormorants. (a) Example of three tracks of birds which left the colony within the same 4‐hr interval (white star: Siniya colony; circles: dives). Birds 27 and 31 departed to sea 3.5 hr after bird 29. (b) Short‐term foraging area consistency expressed as the distance between the barycenter of dive positions in relation to the difference in the time of departure from land between trips of birds which left the colony within 4 hr of one another (black circles, *y *=* *6.17 × (1 − 0.18^*x*^), *R* ^2^ = .20, *P* = .016, *n* = 28) and frequency distribution of trip time differences (gray vertical bars, *n* = 28). (c) Long‐term foraging area consistency expressed as the distance between the barycenter of dive positions of successive trips on the same day (day 1) or on days 2–4 relative to the first trip of day 1 (*n* = 405 distance combinations from 18 birds). *P*‐values indicate statistical differences between days 2, 3, or 4 relative to the first trip of day 1 (***P < .001)

The distance in the barycenter of foraging dive positions increased from 0.5 to 5 km between birds which left the colony simultaneously (within 5 min of each other) or within 1 hr of each other. This distance increased from 5 to 6 km for a 1–2‐hr difference in departure time and stabilized around 6 km for a 2–4‐hr difference in departure time (Figure 4a,b). Birds carried out a maximum of two trips daily (Table 1). We calculated the distance between the barycenter of foraging dive positions of successive trips in the same day and the distance between the barycenter of foraging dive positions of the first trip on day 1 and the following days (Figure 4c). There was no statistical difference in distances between foraging area positions of trips belonging to the same birds or to different birds (*df* = 386, *t* = −0.17, *P* = .865). The average distance between barycenter of dive positions of first and second trips on the same day was 10 km. This distance increased to 32 km between the first trip of day 1 and day 2 (*df* = 384, *t* = 9.25, *P* < .001), 47 km between the first trip of day 1 and day 3 (*df* = 384, *t* = 12.24, *P* < .001), and 52 km between the first trip of day 1 and day 4 (*df* = 384, *t* = 10.41, *P* < .001).

### 3.3 Foraging interactions

**Figure 5:**
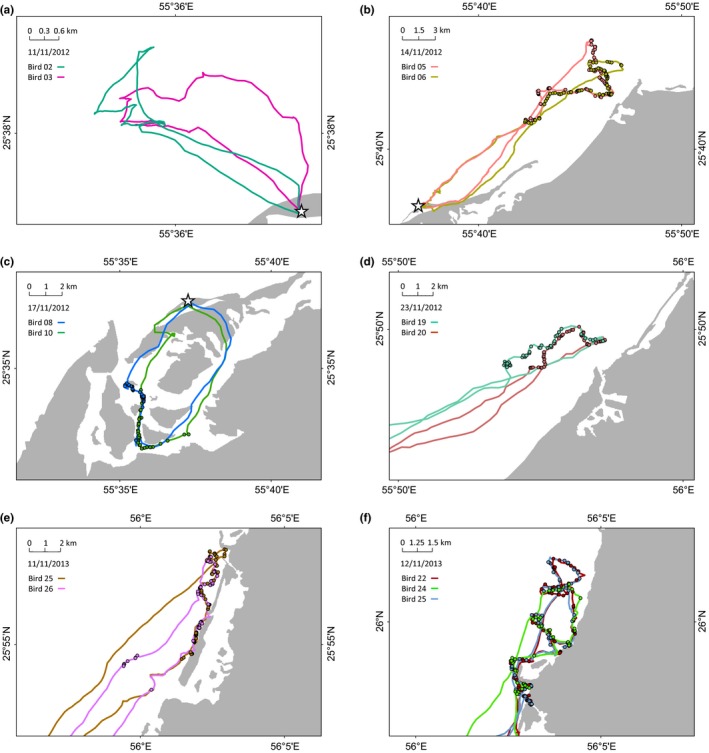
Examples of foraging tracks of Socotra cormorants that are closely associated in space and time (white star: Siniya colony; filled circles: dives). Intraflock association between two birds (a–e) or three birds (f); birds 2 and 3 in (a) did not dive

**Figure 6:**
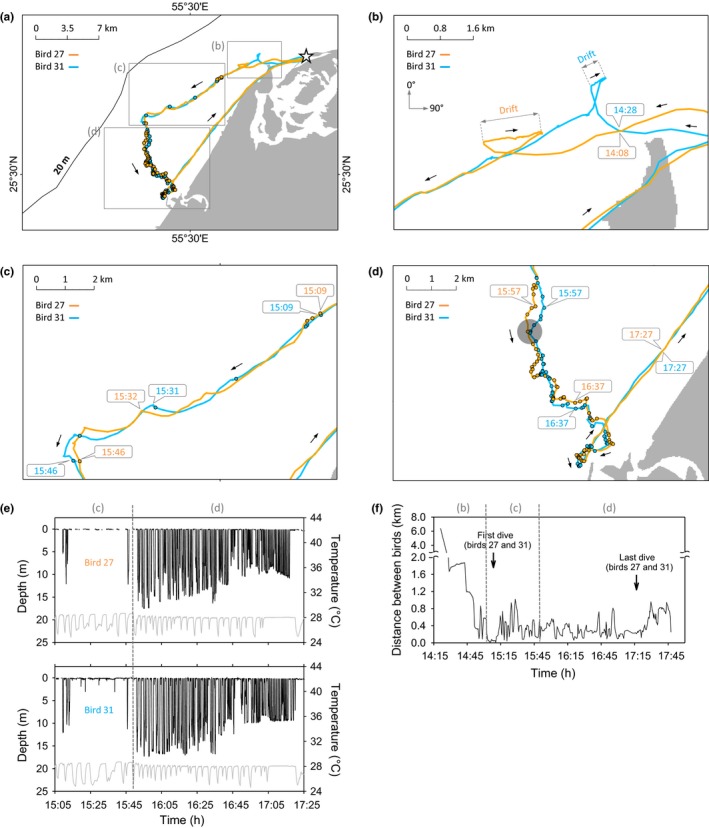
Analysis of an intraflock association (presented in Figure 4a) between Socotra cormorants (white star: Siniya colony; filled circles: dives). (a) Path taken by the flock: the journey comprised 1) a southwesterly outbound phase involving drifting at the sea surface (b) followed by prospective foraging (c), 2) a southeasterly foraging phase with serial diving (d), and 3) a northeasterly inbound phase. (b) Raft formation: the flock, constituted by one or several rafts, was joined separately by birds 27 and 31. Birds drifted at the sea surface following a northeasterly coastal current (birds 27 and 31 drifted for 43 and 12 min, with a speed of 0.41 and 0.39 m/s and a bearing of 80° and 60°, respectively). (c) Prospective group foraging: Birds 27 and 31 closely associated within the flock. After a short dive bout (starting 15:09), this phase was characterized by flights alternating with short periods at the sea surface (e). Surface periods were presumably intended for exploration by birds of the water column for fish, either visually by submergence of the head or by shallow diving, as in bird 31. (d) Intensive group foraging: birds dived serially within one long bout. The dark circle represents the hypothesized maximum width of the flock at the sea surface (f). After the last dive, birds flew back to the colony simultaneously at an average speed of 44 km/hr (no stopover). (e) Dive profiles of birds during the prospective (c) and intensive (d) group‐foraging phases. Temperature profiles (gray lines) indicate the position of flights (for detail, see Figure S2). Synchronous diving was not detected in these two study birds, although they clearly dived in a coordinated manner. They carried out a comparable number of dives (94 and 115 dives by birds 27 and 31, respectively), which became shallower as the flock progressed toward the shore, suggestive of benthic diving, in accordance with bird dive depth and local bathymetry (a). Benthic dives were occasionally interspersed with shallower pelagic dives. Furthermore, birds undertook a comparable number of short flights between dives: 23 and 27 flights by birds 27 and 31, respectively, each lasting 0.5 ± 0.5 min. (f) Distance between birds within the flock: between the first and the last dive of the trip, birds were distant by 341 ± 196 m on average (range: 27–1,030 m)

**Figure 7:**
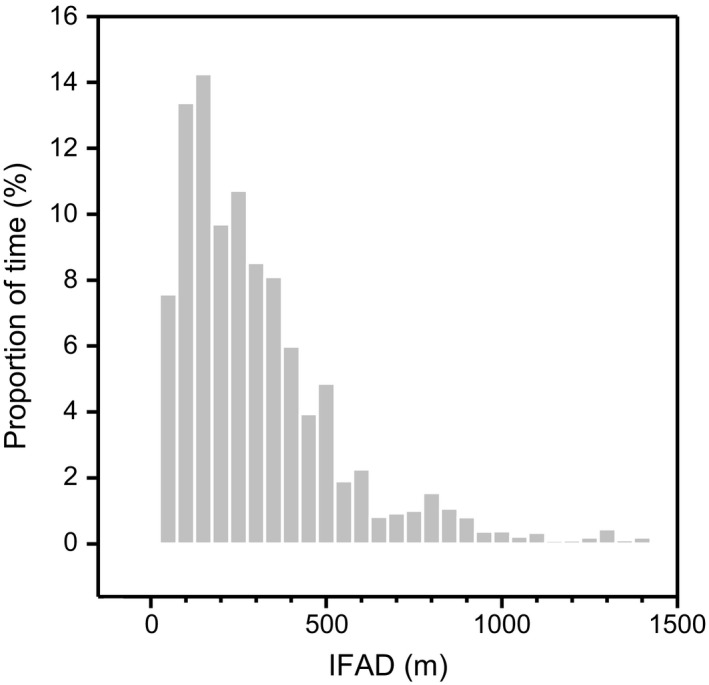
Proportion of time spent by Socotra cormorants in different classes of intraflock association distances (IFADs). Proportion was calculated out of a total of 17.3 hr of association between individuals from eight “pairs” of birds (for detail, see Figure S6). The 25, 50, 75, and 90 percentiles of distribution correspond to 109, 227, 372, and 591 m, respectively

Birds that departed to sea within a few minutes or hours of each other foraged over the same grounds, sometimes associating closely with each other in space and time (Figures 5 and 6). The mean intraflock association distance (IFAD) for eight bird “pairs” (1,784 distances calculated) was 285 ± 235 m (range 8–1,380 m; Figure 7). Maximum flock width on the sea surface was therefore considered to be ca. 1.4 km. However, 87% of the time spent by birds in the flock was spent in IFADs <500 m, suggesting the core of flocks was no wider than 0.5 km. Intraflock associations lasted for an average of 2.2 ± 0.8 hr. During group foraging, birds interspersed their dives with many foraging flights (flights occurring between the first and the last dive of a trip): on average one foraging flight for every 3.7 ± 2.2 dives. Foraging flights lasted 33 ± 29 s (Figure S7).

### 3.4 Diving behavior

**Figure 8:**
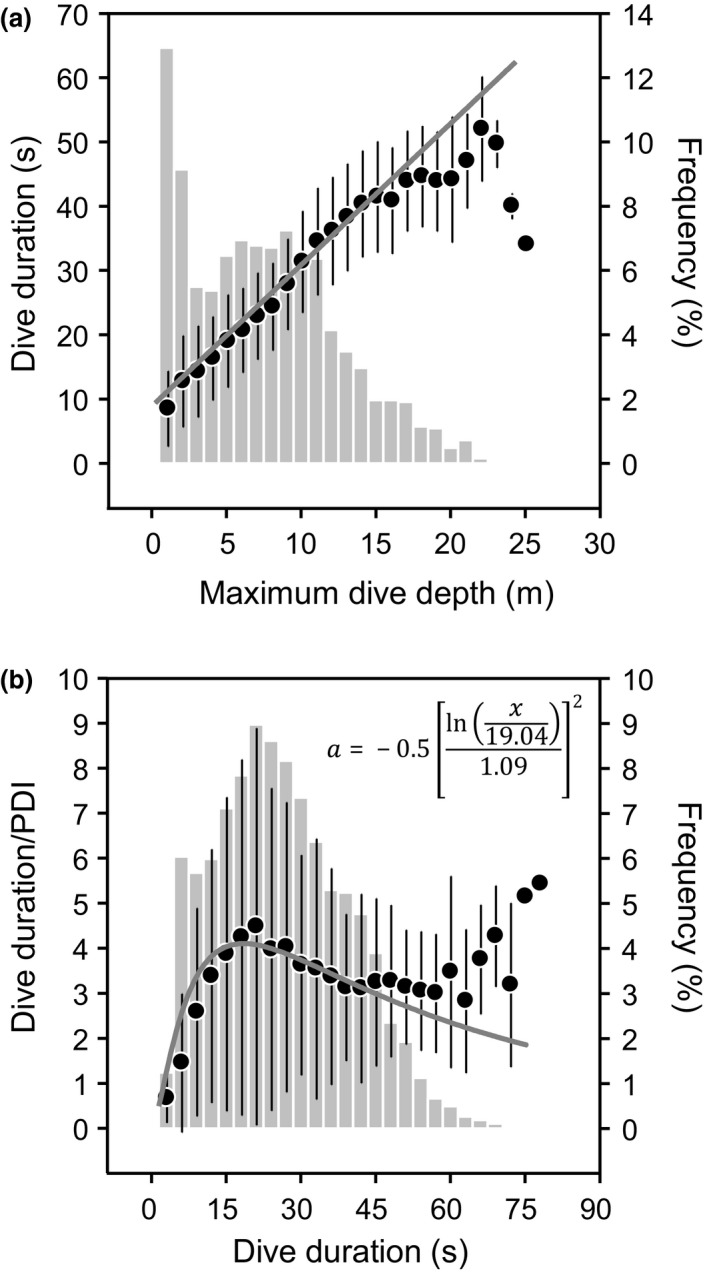
Diving behavior of Socotra cormorants. (a) Relationship between dive duration and maximum dive depth (black circles, *y *=* *2.20*x* + 8.84, *R* ^2^ = .69, *P* < .0001, *n* = 5,225) and frequency distribution of dive depth (gray vertical bars, *n* = 5,225). (b) Relationship between dive duration/postdive interval (PDI) and dive duration (black circles, *y *=* *4.11e^*a*^, *R* ^2^ = .06, *P* < .0001, *n* = 5,205) and frequency distribution of dive duration (gray vertical bars, *n* = 5,225)

Dive parameters are summarized in Table 2. Birds dived to shallow depths, with 22% and 92% of dives carried out to depths ≤2 and 15 m, respectively (Figure 8a). Accordingly, dive durations were short, with 67% of dives ≤30 s and 93% ≤45 s (Figure 8b). Dive duration increased linearly with maximum dive depth (Figure 8a). Frequency distribution of dive durations peaked for dives lasting 15–30 s and then decreased. Concomitantly, the dive duration/postdive interval ratio peaked for dives lasting 15–30 s, before decreasing (Figure 8b). Birds therefore favored a diving behavior that maximized the proportion of time spent underwater relative to the proportion spent at the surface. Dive profiles were parabolic (9.1%), V‐shaped (27.2%), irregular (28.2%), and flat‐bottomed (35.4%). No sign of synchronous diving was detected. Average maximum water temperature was 27.5 ± 0.9°C (Figure S8).

**Table 2:** Summary of dive parameters of Socotra cormorants breeding on Siniya Island (*n* = 5,525 dives)

	Mean	*SD*	Min.	Max.
Max. dive depth (m)	6.9	5.0	0.2	24.3
Dive duration (s)	24.1	13.2	2	76
Descent duration (s)	6.2	4.0	0	26
Descent rate (m/s)	0.93	0.35	0.01	3.07
Bottom duration (s)	12.2	8.7	0	50
Ascent duration (s)	5.7	3.8	0	25
Ascent rate (m/s)	1.04	0.43	0.01	2.94
Postdive interval (PDI, s)	12.5	14.4	0	100
Dive duration/PDI	3.6	3.5	0.03	59
Max. dive temperature (°C)	27.5	0.9	23.8	29.5

## 4 Discussion

To understand the benefits of group foraging in seabirds, we need to understand how they forage, in particular what strategies they use to locate and secure their prey. Using GPS and temperature–depth recorders deployed simultaneously on breeding individuals, we successfully identified some of the processes underlying group foraging in Socotra cormorants. Results suggest that the benefits stemming from this behavior should be increased likelihood of prey detection and capture.

### 4.1 Evidence of group foraging

Because Socotra cormorant study nests were dispersed across the colony, study birds were not neighbors and likely not related. We therefore considered that the distance between nests had no influence on the probability that two study birds had of leaving the colony together and, consequently, of commuting toward the same foraging grounds (Berlincourt & Arnould, 2014). Assuming that every nesting bird in the colony went to sea once a day, that partners alternated nest attendance, and that the number of breeding pairs in the colony was ca 33,000 (S.B. Muzaffar, unpublished data), we estimated the number of birds at sea at any time to be around 33,000 (excluding nonbreeders). Hence, assuming there is more than one suitable foraging ground in the area at any given moment, the theoretical probability (*P*) that two study birds that were at sea at the same time would visit the same grounds by chance was very low (*P *= [2/33,000] × [1/32,999] = 1.8 × 10^−9^). Yet, this probability was virtually 100% in our study, as shown by the average distance between the foraging area positions of all birds departing from the colony together (0.5 km, Figure 4a,b) or within 1 hr of each other (5 km, Figure 4b). This was further illustrated by the overlap between tracks of birds that were at sea at the same time (Figures 5 and 6). These results demonstrate three things: (1) Birds aggregated at sea and foraged together, most likely in one large flock (up to 33,000 individuals, excluding nonbreeders), (2) they commuted to foraging grounds using social information, and (3) they foraged roughly over the same area throughout the day. In group‐foraging Socotra cormorants, the whole colony can be seen as working together as a single social unit.

### 4.2 Advantages of group foraging for prey detection

Adequate foraging grounds of Socotra cormorants were likely discovered through the effect of the multitude of eyes scanning the water surface (Fernández‐Juricic, Erichsen, & Kacelnik, 2004). Opportunistic field observations show that in the morning, birds aggregate on the beach near the colony until they eventually depart *en masse*. Such groups comprise members of both sexes, contrary to some cormorant species where foraging groups can be sex specific (Cook et al., 2013). A massive raft comprising thousands of birds floating on the water surface may also be found close to the colony. This raft eventually takes off as one large flock and heads down the coast in loose formation. The flock is made up of scattered multiple lines of 10–50 birds flying in half‐V formations (an asymmetrical version of the V formation) often just above the water surface presumably in order to reduce flight expenditure (Tanida, 2001; Portugal et al., 2014; Figure 1c). It is probable these individual streams of birds scan different parts of the water surface and recruit other birds of the flock through local enhancement when food is discovered (Bairos‐Novak et al., 2015). In such a system, an individual's connectivity to others (social network) is likely to be a crucial part of the process of relaying information (Aplin, Farine, Morand‐Ferron, & Sheldon, 2012). Waters of the Arabian Gulf are relatively transparent, so spotting a school of fish in shallow waters from the air would seem relatively easy at close range. While commuting, Socotra cormorants may occasionally prospect the deeper part of the water column by landing, diving, and taking off again (Figure 6c). Seabirds may also rely on the presence of other species to locate fish schools (Tremblay et al., 2014). Other abundant local predators that depend on the same resource include the finless porpoise (*Neophocaena phocaenoides*), the Indo‐Pacific humpback (*Sousa chinensis*), and Indo‐Pacific bottlenose (*Tursiops aduncus*) dolphins and the lesser crested (*Thalasseus bengalensis*), bridled (*Sterna anaethetus*), and white‐cheeked (*Sterna repressa*) terns (Behrouzi‐Rad, 2013; Braulik et al., 2010).

Searching behavior during the outbound phase translated into relatively slower bird flight, more frequent stopovers, and a higher proportion of time spent at the sea surface than during the inbound phase. As a consequence, Socotra cormorants had a higher average velocity during the inbound phase (Figure 3b). Newly departing birds most likely retraced the location of the foraging flock at sea based on the bearing of incoming birds (e.g., Greene, 1987; Machovsky‐Capuska, Hauber, Libby, Amiot, & Raubenheimer, 2014; Thiebault, Mullers, Pistorius, Meza‐Torres, et al., 2014). During the noon shift, opportunistic observations from within the colony suggest that leaving and incoming birds departed and returned in groups of 10–50 individuals over period of about 2 hr. Bird average velocity during the outbound phase increased over the day (Figure 3c), lending support to the hypothesis that Socotra cormorants are slower at reaching foraging grounds in the early morning, when prey is still not located, than later during the day, when birds are informed on the location of the food patch by returning birds. Hence, the foraging flock, once it is established at sea in the morning, functions as a focal point, attracting and losing birds throughout the day, suggesting some degree of fission–fusion dynamics between individuals (Aureli et al. 2008). The core position of the flock shifted somewhat over time (by up to 10 km, Figure 4a,b), with birds presumably keeping track of the fish school through the effect of numbers. It is unclear whether there is a link between the position of foraging grounds in the morning and on the following days. The distance between the two increased over time, pointing to some element of memory guiding the choice of morning foraging area. However, if present, the role of this memory seems to disappear entirely after 2 days (Figure 4c), suggesting a relatively short lifetime of local productive areas.

### 4.3 Advantages of group foraging for prey capture

Socotra cormorants target anchovy, bluestripe herring, and African sailfin flying fish in the eastern Arabian Gulf (Muzaffar et al., 2016). Assuming they ate mainly anchovy (Muzaffar et al., 2016), the total fish consumption of Socotra cormorants from Siniya Island (including nonbreeders) during a breeding season amounted to 5,078 tonnes (range: 3,506–7,263 tonnes) or 47 tonnes per day on average (range: 33–68 tonnes) (Appendix S1). Considering the geographic consistency of the foraging area throughout the day, these results imply that the fish school (or schools) exploited by cormorants on a daily basis are of considerable size. Evidence suggests that anchovies, sardines, and herrings migrate slowly during the breeding period in a roughly east‐to‐west direction along the United Arab Emirates coastline (Ministry of Climate and Environment, unpublished data). Thus, during the breeding period, schools of small forage fish would be consistently abundant within the foraging range of Socotra cormorants.

If Socotra cormorants split school formations and disperse the fish too much, foraging is no longer cost‐efficient (Berlincourt & Arnould, 2014). Cormorants must therefore concentrate fish schools, something which is facilitated by the effect of bird numbers (Allee, Emerson, Park, Park, & Schmidt, 1949; Ryan, Edwards, & Pichegru, 2012). The proximity of the seafloor (Figure 2c) can also help herd the fish. In view of the diversity of dive profiles, cormorants used the entire water column, carrying out dives pelagically and epibenthically (Cook et al., 2012), while fish were trapped between the surface and the nearby seafloor. In the example in Figure 6, birds moved progressively toward the shoreline, pushing schools into shallower and shallower waters. At sea, Socotra cormorants carried out one foraging flight for every four dives on average (Figure S2), meaning they were constantly on the move, even if these flights were short (30 s on average). By comparison, a solitary benthic foraging species like the Crozet shag (*Leucocarbo melanogenis*) carries out one foraging flight for every 33 dives (Cook, Cherel, & Tremblay, 2006). In view of their average flight speed (45 km/hr), these short hops allowed Socotra cormorants to move forward by 400 m each time, thus constantly keeping up with the flock (Figure 7). This behavior can be compared to a conveyor belt, with birds continuously catching up with the moving flock and overtaking birds that are still under water (van Eerden & Voslamber, 1995). Schools of small pelagic fish typically travel at 0.4–1.9 m/s (e.g., Misund, Fernö, Pitcher, & Totland, 1998; Misund et al., 2003; Peraltilla & Bertrand, 2014). In comparison, mean instantaneous ground speed of Socotra cormorants during the foraging phase was 2.1 ± 0.3 m/s, matching the speed of fish schools. Fish may also use the coastal current for some form of passive transport. In the example in Figure 4a, the time lag between the position of dives during the morning and the afternoon trips would translate into prey moving at 0.3 m/s, which is close to the 0.4 m/s current calculated that same day using birds as drifter buoys (Figure 6b) and in accordance with the known direction of the coastal current in the area (Pous, Lazure, & Carton, 2015). Underwater, cormorants swim at 1.5–2.5 m/s (Cook, Kato, Tanaka, Ropert‐Coudert, & Bost, 2010). Fish may increase speed during burst swimming in order to escape a predator; however, they cannot sustain this for long and will quickly get exhausted (van Eerden & Voslamber, 1995).

Once fish have been aggregated, however, they are better protected from Socotra cormorants due to the “predator confusion effect,” which makes it difficult for the predator to visually pick out individual prey within the large swirling mass of flashing fish (Milinski & Heller, 1978) and to the “many‐eyes effect” (Lima, 1995), which increases the speed of response of prey to predator attack via propagation of escape waves across the school (e.g., Radakov, 1973; Axelsen, Anker‐Nilson, Fosum, Kvamme, & Nottestad, 2001; Gerlotto et al. 2006; Herbert‐Read, Buhl, Feng, Ward, & Sumpter, 2015; Rieucau, Holmina, Castilloc, Couzind, & Handegarda, 2016). Birds must therefore disorganize the fish school sufficiently to reduce its coordinated antipredator behavior. Socotra cormorants targeted dives lasting around 15–30 s, a duration range yielding the highest values of dive‐to‐surface ratio (Figure 8b). Such a strategy is termed “optimal breathing” (Cook, Lescroël, Tremblay, & Bost, 2008) and is related to the uptake rate of oxygen in birds during postdive intervals, which should have been fastest precisely after dives lasting around 15–30 s. Such dives depleted only the respiratory tract oxygen, but not the blood hemoglobin and skeletal muscle myoglobin stores, which take longer to replenish (Cook et al., 2008). As a result, Socotra cormorants maximized time foraging underwater proportional to time at the surface, which is time lost to foraging. Given an average dive‐to‐pause ratio of 3.6, the proportion of birds underwater was 72%. This means that for an actively foraging flock of 44,550 birds (composed of half of the breeding adults and half of the nonbreeders from Siniya, Appendix S1) and assuming a flock diameter of 500 m (Figure 7), the average density of birds underwater was 0.16 birds/m^2^. Although this result corresponds to a situation of maximum possible number of birds in the flock, it illustrates the magnitude of the phenomenon. It suggests that fish schools were under continuous harassment by birds, which would have the effect of disorganizing school cohesiveness and facilitating prey capture (Wilson et al., 1987). Hence, in this case, the constant successive attacks of birds likely increased individual prey intake rate compared to that acquired through solitary foraging (Thiebault et al., 2016). In view of the dive profiles (Figure 6e), birds did not appear to dive in synchrony (Saino, Fasola, & Waiyaki, 1995). Synchronous diving necessitates visual coordination between divers, which would be difficult in a group of such size. In penguins, synchronous diving occurs in small groups (Berlincourt & Arnould, 2014) but disappears when the group becomes larger, as birds lose contact under water (Wilson et al., 1986; but see Ryan et al., 2012).

## 5 Conclusions

Our data lend support to the hypothesis that aggregation in seabirds leads to a faster discovery of food patches and, through local enhancement, to an efficient transfer of information about patch location (e.g., Boyd et al., 2016; Buckley, 1997; Thiebault, Mullers, Pistorius, & Tremblay, 2014; Weimerskirch, Bertrand, Silva, Marques, & Goya, 2010). Our data also support the hypothesis that prey capture is facilitated during group foraging, via a combination of prey herding and school disorganization (e.g., Berlincourt & Arnould, 2014; Thiebault et al., 2016; Tremblay & Cherel, 1999; Wilson et al., 1987). Field studies of group foraging are challenging, but biologging represents an efficient and cost‐effective solution. Understanding the mechanisms underlying group foraging in seabirds is important not only from an evolutionary perspective, but also from a conservation perspective. If forming large groups is important to forage successfully, seabirds that have undergone a decline in numbers may face difficulty in securing prey efficiently, thus further increasing the threat to their species (Ryan et al., 2012).

## Conflict of Interest

None declared.
