# Supplementary material for: Group foraging in Socotra cormorants: A biologging approach to the study of a complex behavior
Source: Ecol Evol. 2017 Feb 26;7(7):2025–38. doi: 10.1002/ece3.2750 (PMC5383476; doi:10.1002/ece3.2750)
Supplement: Supplementary file 1 [file ECE3-7-2025-s001.docx]

**Online Additional Supporting Information**

**Title**: Group foraging in Socotra cormorants: a bio-logging approach to the study of a complex behaviour

**Authors**: Timothée R Cook, Rob Gubiani, Peter G Ryan, Sabir B Muzaffar

**Journal**: Ecology & Evolution

**Table S1.** Sex, nest content and morphological parameters of Socotra cormorants breeding on Siniya Island. Individuals were sexed by molecular sexing on feathers plucked from birds. Birds 19 and 20 were not weighed or sampled for feathers and were thus sexed using a discriminant function analysis performed in Statistica 8.0 (Stat-Soft, Inc.) on morphological variables (excluding body mass) of 22 birds of known sex (5 males and 17 females).

| Year | Bird | Sex | Eggs | Chicks* | Mass (g) | Tarsus (mm) | Wing (mm) | Culmen (mm) | Bill Width (mm) | Bill Depth (mm) | Head + Beak (mm) |
| --- | --- | --- | --- | --- | --- | --- | --- | --- | --- | --- | --- |
| 2012 | 1 | F | 4 | 0 | 1370 | 73.7 | 281 | 65.4 | 12.6 | 13.2 | 131.3 |
| 2012 | 2 | F | 1 | 1 | 1510 | 74.1 | 280 | 65.1 | 11.9 | 14.1 | 134.2 |
| 2012 | 3 | F | 2 | 1 | 1365 | 72.4 | 275 | 65.4 | 12.4 | 14 | 129.6 |
| 2012 | 5 | F | 1 | 2 | 1550 | 72.6 | 289 | 68.6 | 12.2 | 15 | 136.2 |
| 2012 | 6 | F | 2 | 2 | 1300 | 70.1 | 268 | 65.7 | 12.1 | 13.7 | 126.7 |
| 2012 | 7 | F | 3 | 1 | 1320 | 70.8 | 279 | 68.9 | 12.9 | 13.4 | 132.8 |
| 2012 | 8 | F | 2 | 2 | 1550 | 73.8 | 283 | 71.4 | 13 | 14.5 | 134.2 |
| 2012 | 10 | M | 3 | 1 | 1750 | 73.9 | 295 | 72.6 | 13.8 | 16.2 | 140.8 |
| 2012 | 13 | M | 0 | 2 | 1760 | 79.1 | 308 | 75.5 | 14 | 17.1 | 145.5 |
| 2012 | 19 | M | 3 | 1 | − | 71.1 | 289 | 65.6 | 11.6 | 17 | 132.1 |
| 2012 | 20 | M | 3 | 1 | − | 70.9 | 288 | 64.2 | 11.9 | 16.6 | 135.5 |
| 2013 | 22 | M | 1 | 2 | 1610 | 76.3 | 293 | 67.1 | 13.2 | 15.3 | 136 |
| 2013 | 23 | F | 4 | 0 | 1460 | 72.4 | 286 | 61.7 | 11.5 | 14 | 133.1 |
| 2013 | 24 | M | 3 | 1 | 1820 | 73.3 | 297 | 72.7 | 13.3 | 15.5 | 142.2 |
| 2013 | 25 | F | 4 | 0 | 1350 | 71.4 | 275 | 69.3 | 12 | 14.8 | 131.2 |
| 2013 | 26 | F | 3 | 1 | 1450 | 72.7 | 283 | 65.2 | 12 | 14.5 | 131.2 |
| 2013 | 27 | F | 1 | 2 | 1450 | 68.7 | 276 | 65.1 | 11.3 | 13.4 | 132.2 |
| 2013 | 29 | F | 2 | 1 | 1605 | 71.4 | 279 | 67.1 | 12.1 | 14.4 | 134.1 |
| 2013 | 31 | F | 2 | 1 | 1620 | 76.3 | 286 | 67.1 | 11.4 | 15 | 133.6 |
| 2013 | 36 | F | 3 | 1 | 1610 | 74 | 285 | 67.7 | 12.3 | 14.7 | 136.8 |

*Hatchlings.

**Table S2.** Individual general foraging and dive parameters of Socotra cormorants breeding on Siniya Island.

| Year | Bird | Days at sea | Trips at sea | Dives | Dives per day | Dives per trip | Max. dive depth (m) | Mean dive depth (m) | Max. dive duration (s) | Mean dive duration (s) |
| --- | --- | --- | --- | --- | --- | --- | --- | --- | --- | --- |
| 2012 | 1 | 2 | 2 | 109 | 55 ± 5 | 54 ± 4 | 22.5 | 6.6 ± 4.9 | 59 | 23.3 ± 11.8 |
| 2012 | 2 | 1 | 3 | 104 | 104 | 52 ± 6 | 21.6 | 10.4 ± 5.7 | 48 | 25.1 ± 11.9 |
| 2012 | 3 | 1 | 3 | 49 | 49 | 24 ± 10 | 24.3 | 11.7 ± 6.3 | 52 | 29.6 ± 11.3 |
| 2012 | 5 | 1 | 1 | 105 | 105 | 105 | 13.6 | 10.2 ± 1.9 | 61 | 39.1 ± 10.1 |
| 2012 | 6 | 1 | 1 | 123 | 123 | 123 | 13.1 | 9.3 ± 2.5 | 52 | 30.1 ± 10.1 |
| 2012 | 7 | 1 | 1 | 82 | 82 | 82 | 12.2 | 1.7 ± 2.1 | 33 | 9 ± 6.8 |
| 2012 | 8 | 3 | 4 | 440 | 147 ± 63 | 110 ± 26 | 14.7 | 5.7 ± 4.1 | 53 | 21.5 ± 9.7 |
| 2012 | 10 | 3 | 4 | 576 | 192 ± 100 | 144 ± 77 | 21 | 3.5 ± 4.9 | 64 | 19.4 ± 10.4 |
| 2012 | 13 | 1 | 1 | 63 | 63 | 63 | 11.8 | 7.3 ± 3.7 | 61 | 27.7 ± 13.1 |
| 2012 | 19 | 4 | 4 | 616 | 154 ± 44 | 154 ± 44 | 12.1 | 5.9 ± 2.9 | 44 | 17.5 ± 8.3 |
| 2012 | 20 | 4 | 4 | 630 | 158 ± 49 | 157 ± 49 | 19.6 | 5.6 ± 3.3 | 71 | 21.6 ± 11.4 |
| 2013 | 22 | 3 | 3 | 421 | 140 ± 11 | 140 ± 11 | 22.1 | 6.6 ± 4.2 | 53 | 23.5 ± 11 |
| 2013 | 23 | 2 | 3 | 274 | 137 ± 35 | 91 ± 12 | 19 | 6.1 ± 3.6 | 48 | 18.5 ± 9.2 |
| 2013 | 24 | 3 | 3 | 226 | 75 ± 18 | 75 ± 18 | 20.3 | 9.1 ± 4.3 | 60 | 30.9 ± 12.1 |
| 2013 | 25 | 2 | 2 | 263 | 132 ± 38 | 131 ± 37 | 22.5 | 7.1 ± 5.9 | 60 | 23.3 ± 14.2 |
| 2013 | 26 | 3 | 5 | 319 | 106 ± 33 | 80 ± 23 | 18.2 | 8.2 ± 5.6 | 64 | 29.2 ± 15.4 |
| 2013 | 27 | 2 | 3 | 191 | 96 ± 5 | 64 ± 44 | 17.1 | 7.1 ± 4.7 | 59 | 26.7 ± 12.5 |
| 2013 | 29 | 1 | 1 | 86 | 86 | 86 | 17.9 | 12.1 ± 4.6 | 63 | 36.4 ± 14.3 |
| 2013 | 31 | 3 | 4 | 333 | 111 ± 30 | 111 ± 30 | 21.2 | 11.6 ± 6 | 76 | 34.9 ± 17.3 |
| 2013 | 36 | 2 | 2 | 215 | 108 ± 70 | 107 ± 69 | 19.1 | 9.7 ± 4.1 | 67 | 32.2 ± 13.1 |

**Table S3.** Individual foraging trip and time-budget parameters of Socotra cormorants breeding on Siniya Island.

| Year | Bird | Trips per day | Max. trip duration (h) | Mean trip duration (h) | Max. daily time flying (h) | Mean daily time flying (h) | Max. daily time at the surface (h) | Mean daily time at the surface (h) | Max. daily time diving (h) | Mean daily time diving (h) |
| --- | --- | --- | --- | --- | --- | --- | --- | --- | --- | --- |
| 2012 | 1 | 1 | 2.1 | 2.1 | 0.6 | 0.6 ± 0 | 1.1 | 1.1 ± 0 | 0.4 | 0.4 ± 0 |
| 2012 | 2 | 1.5 ± 0.5 | 1.3 | 1.3 | − | − | − | − | − | − |
| 2012 | 3 | 1.5 ± 0.5 | 1.5 | 1.5 | − | − | − | − | − | − |
| 2012 | 5 | 1 | 3.6 | 3.6 | 1.1 | 1.1 ± 0 | 1.3 | 1.3 ± 0 | 1.1 | 1.1 ± 0 |
| 2012 | 6 | 1 | 2.9 | 2.9 | 1.2 | 1.2 ± 0 | 0.7 | 0.7 ± 0 | 1.0 | 1.0 ± 0 |
| 2012 | 7 | 1 | 6.7 | 6.7 | 3.1 | 3.1 ± 0 | 3.3 | 3.3 ± 0 | 0.2 | 0.2 ± 0 |
| 2012 | 8 | 1.3 ± 0.5 | 4.3 | 2.2 ± 1.3 | 1.6 | 0.9 ± 0.6 | 1.8 | 1.2 ± 0.4 | 1.1 | 0.9 ± 0.2 |
| 2012 | 10 | 1.3 ± 0.5 | 4.1 | 3.1 ± 0.8 | 2.2 | 1.3 ± 0.7 | 3.5 | 1.8 ± 1.2 | 1.3 | 1.0 ± 0.3 |
| 2012 | 13 | 1 | 3.7 | 3.7 | 2.4 | 2.4 ± 0 | 0.9 | 0.9 ± 0 | 0.5 | 0.5 ± 0 |
| 2012 | 19 | 1 | 4.3 | 3.5 ± 0.5 | 1.9 | 1.7 ± 0.1 | 1.5 | 1 ± 0.3 | 1.0 | 0.7 ± 0.1 |
| 2012 | 20 | 1 | 7.4 | 4.8 ± 1.9 | 3.1 | 2.1 ± 0.6 | 3.6 | 2.2 ± 1.3 | 1.8 | 0.9 ± 0.5 |
| 2013 | 22 | 1 | 6.2 | 5.3 ± 0.8 | 3.1 | 2.5 ± 0.5 | 2.2 | 1.9 ± 0.3 | 1.1 | 0.9 ± 0.1 |
| 2013 | 23 | 1.5 ± 0.5 | 5.9 | 4.5 ± 1 | 4.6 | 3.9 ± 0.7 | 2.3 | 2.2 ± 0.1 | 1.0 | 0.7 ± 0.3 |
| 2013 | 24 | 1 | 5.5 | 4.3 ± 0.9 | 2.8 | 2.3 ± 0.4 | 2.1 | 1.4 ± 0.5 | 0.8 | 0.6 ± 0.1 |
| 2013 | 25 | 1 | 5.6 | 5.5 ± 0.1 | 3.2 | 2.9 ± 0.3 | 2 | 1.8 ± 0.2 | 0.9 | 0.8 ± 0 |
| 2013 | 26 | 1.2 ± 0.4 | 5.2 | 3.6 ± 1.6 | 2.7 | 2.1 ± 0.8 | 2.2 | 1.9 ± 0.2 | 1.3 | 0.9 ± 0.3 |
| 2013 | 27 | 1.5 ± 0.5 | 4.4 | 3.5 ± 0.9 | 2.6 | 2.3 ± 0.4 | 3.3 | 2.3 ± 1.0 | 0.9 | 0.7 ± 0.2 |
| 2013 | 29 | 1 | 3.4 | 3.4 | 1.4 | 1.4 ± 0 | 1.2 | 1.2 ± 0 | 0.9 | 0.9 ± 0 |
| 2013 | 31 | 1 | 5.9 | 3.7 ± 1.6 | 2.5 | 2 ± 0.4 | 2.4 | 2 ± 0.4 | 1.3 | 1.1 ± 0.1 |
| 2013 | 36 | 1 | 3.0 | 2.6 ± 0.4 | 1 | 0.8 ± 0.2 | 1.2 | 0.9 ± 0.3 | 1.5 | 1.0 ± 0.5 |

**Table S4.** Individual foraging trip parameters of Socotra cormorants breeding on Siniya Island: schedule, distance and path length.

| Year | Bird | Time first daily trip (h:min) | Time last daily trip (h:min) | Max. distance (km) | Mean max. distance (km) | Max. path length (km) | Mean path length (km) |
| --- | --- | --- | --- | --- | --- | --- | --- |
| 2012 | 1 | 14:40 ± 00:00 | 16:44 ± 00:00 | 6.2 | 6.2 | 27 | 27 ± 0 |
| 2012 | 2 | 11:09 ± 04:26 | 16:52 ± 00:00 | 14.1 | 9.1 ± 4.9 | 16.2 | 16.2 ± 0 |
| 2012 | 3 | 11:48 ± 04:09 | 17:30 ± 00:00 | 3.9 | 3.9 | 12.1 | 12.1 ± 0 |
| 2012 | 5 | 09:58 ± 00:00 | 13:32 ± 00:00 | 20.9 | 20.9 | 56.2 | 56.2 ± 0 |
| 2012 | 6 | 09:55 ± 00:00 | 12:49 ± 00:00 | 21.0 | 21 | 56.8 | 56.8 ± 0 |
| 2012 | 7 | 07:50 ± 00:00 | 14:29 ± 00:00 | 55.7 | 55.7 | 154.3 | 154.3 ± 0 |
| 2012 | 8 | 10:26 ± 01:06 | 12:40 ± 01:32 | 28.5 | 12.3 ± 9.5 | 78.3 | 35.2 ± 25.1 |
| 2012 | 10 | 10:48 ± 01:59 | 13:55 ± 02:44 | 44.6 | 17.4 ± 15.8 | 108.6 | 48.1 ± 35.7 |
| 2012 | 13 | 11:06 ± 00:00 | 14:46 ± 00:00 | 42.1 | 42.1 | 98.6 | 98.6 ± 0 |
| 2012 | 19 | 11:44 ± 00:30 | 15:15 ± 00:48 | 40.9 | 37.4 ± 5.6 | 95.9 | 88.1 ± 8.7 |
| 2012 | 20 | 10:24 ± 02:44 | 15:15 ± 01:01 | 57.0 | 39.3 ± 11.3 | 145.2 | 103.6 ± 28.7 |
| 2013 | 22 | 11:37 ± 00:26 | 16:56 ± 01:06 | 63.9 | 54.5 ± 7.6 | 157.1 | 129.7 ± 20.9 |
| 2013 | 23 | 09:08 ± 03:49 | 13:39 ± 03:21 | 63.8 | 54.2 ± 7 | 146.9 | 121.5 ± 18.6 |
| 2013 | 24 | 11:34 ± 00:47 | 15:54 ± 01:34 | 63.2 | 52.9 ± 7.5 | 145.6 | 119.5 ± 18.5 |
| 2013 | 25 | 12:42 ± 00:01 | 18:14 ± 00:06 | 63.9 | 61.2 ± 2.8 | 152.2 | 140.7 ± 11.5 |
| 2013 | 26 | 12:12 ± 03:01 | 15:50 ± 02:52 | 58.2 | 29.1 ± 23.7 | 134.9 | 72 ± 50.4 |
| 2013 | 27 | 11:51 ± 01:39 | 15:22 ± 02:15 | 58.2 | 31.3 ± 19.2 | 128.3 | 74.3 ± 39.2 |
| 2013 | 29 | 10:36 ± 00:00 | 14:01 ± 00:00 | 25.7 | 25.7 | 63.3 | 63.3 ± 0 |
| 2013 | 31 | 10:20 ± 03:17 | 14:04 ± 02:26 | 51.1 | 27.5 ± 16.6 | 123.1 | 70.6 ± 37.7 |
| 2013 | 36 | 10:51 ± 03:36 | 13:28 ± 04:02 | 18.4 | 13.4 ± 4.9 | 46.4 | 37.4 ± 8.9 |

**Table S5.** Results of linear mixed-effects models testing the effect of year on different foraging parameters of Socotra cormorants breeding on Siniya Island.

| Parameter | df | t | p |
| --- | --- | --- | --- |
| Trip start time | 18 | 0.44 | 0.662 |
| Trip end time | 18 | 1.05 | 0.306 |
| Trip duration | 18 | 1.59 | 0.127 |
| Max. linear distance to colony | 18 | 2.08 | 0.052 |
| Path length | 18 | 1.71 | 0.104 |
| Bearing | 18 | -1.07 | 0.299 |
| Time flying per trip | 16 | 1.24 | 0.233 |
| Time at the sea surface per trip | 16 | -0.39 | 0.670 |
| Time diving per trip | 16 | 0.33 | 0.748 |
| Dives per trip | 18 | -0.59 | 0.570 |
| Max. dive depth | 18 | 1.30 | 0.210 |
| Dive duration | 18 | 1.41 | 0.175 |
| Post-dive interval | 18 | 0.87 | 0.397 |
| Max. water temperature during dive | 18 | -0.39 | 0.694 |

**Fig. S1.** Frequency distribution of instantaneous ground speeds of Socotra cormorants breeding on Siniya Island. Ground speeds were calculated between successive GPS points of 49 foraging trips (n = 26266). The black arrow points to the cut-off speed (15 km.h^-1^) between non-flight (diving or resting at the sea surface) behaviour and flight behaviour.

**Fig. S2.** Examples of dive profiles of Socotra cormorants breeding on Siniya Island. (a) Complete foraging bout of bird n° 36 on 18 November 2013. Dives are visible on the depth plot (black line). Flights between dives are visible on the temperature plot (grey line). (b) Close-up and example of dive profiles and flight events (f): V-shape dives (1), irregular dives (2, 4, 5, 6, 7, 8, 9) and parabolic dives (3). (c) Close-up and example of dive profiles and flight events (f): irregular dives (1, 2, 3) and flat-bottomed dives (4, 5, 6, 7, 8, 9).

**Fig. S3.** Activity rhythms of Socotra cormorants breeding on Siniya Island. Frequency distribution of (a) trip departure time from colony (n = 51), (b) trip return time to colony (n = 49) and (c) trip duration (n = 49).

**Fig. S4.** Frequency distribution of post-dive intervals (n = 5205) of Socotra cormorants breeding on Siniya Island.

**Fig. S5.** Polar histogram of bearing of position of maximum distance reached during each foraging trip relative to colony position (n = 49) for Socotra cormorants breeding on Siniya Island.

**Fig. S6.** Distance at sea between birds as a function of time for Socotra cormorants breeding on Siniya Island. (a) Distance between tracks of birds mapped in Fig. 3b (main text). (b-e) Distance between tracks of birds mapped in Fig. 5b-e, respectively (main text). (f-h) Distance between tracks of birds mapped in Fig. 5f (main text).

**Fig. S7.** Frequency distribution of duration of foraging flights (flights occurring between the first and the last dive of the trip, n = 1549) of Socotra cormorants breeding on Siniya Island.

**Fig. S8.** Frequency distribution of maximum water temperature recorded during each dive (n = 5225) for Socotra cormorants breeding on Siniya Island.

**Appendix S1.** Food intake of Socotra cormorants from Siniya Island during the breeding season.

The amount of fish captured by adult Socotra cormorants can be calculated using a time-energy budget modelling approach (Fort, Porter & Grémillet 2011). Considering the duration (*D*) and metabolism per time unit (*M*) of each activity *k*, and the number of different activities undertaken (*n*), we estimated daily energy expenditure (kJ.day^-1^) as:

$$Daily energy expenditure =\sum_{k=1}^{n} (D_{k}\times M_{k})$$

Considering the prey’s calorific value (*Cp*) and the bird’s assimilation efficiency of the prey (*Ea*), daily energy expenditure (*DEE)* was converted to daily food intake (g.day^-1^) with:

$$Daily food intake= \frac{DEE}{Cp \times Ea}$$

Considering the temperature of the water (*Tw*), the fish specific heat capacity (*HC*), the bird’s body temperature (*Tb*) and the bird’s metabolic efficiency (*Em*), we estimated the energy (kJ) necessary to warm the bird’s fish load (*L*) following Grémillet *et al.* (2003):

$$Food warming energy = \frac{\left( Tb -Tw \right)\times HC \times L}{Em}$$

We calculated expenditure for the incubation, early chick-rearing, late chick-rearing and non-breeding phases. Energetic requirements of immatures were considered to be similar to those of non-breeding adults. During the breeding season, we considered that adults maintained body condition and that their daily food intake was comprised of the amount of fish necessary to sustain brood expenditure in addition to their own expenditure, as derived from their time-activity budget. We estimated chick energetic requirements (kJ) with an allometric equation for seabirds (Visser 2002), where *A* is asymptotic chick mass (g) and *F* is days to fledging:

$$Chick daily metabolizable energy intake = \frac{{(11.09 \times A}^{0.771}) \times(F^{0.747})}{F}$$

We assumed that birds’ activity during the study reflected the activity of Socotra cormorants during non-breeding, incubation and early chick-rearing. During early chick-rearing, birds simply capture more prey to account for the need of chicks. In cormorants, daily time foraging typically increases with clutch mass (Grémillet 1997, Kato *et al.* 2000, Daunt *et al.* 2007). We therefore assumed that Socotra cormorants increased by × 1.5 the daily time at sea between early and late chick-rearing. Parameters used to build the model are presented in Table A1. Outputs of the model are presented in Table A2.

An average of 33000 pairs of Socotra cormorants breed on Siniya Island (Muzaffar, unpublished data). To those 66000 breeders, we considered that immatures (1 year old) and non-breeding adults (2 years old) comprised 23% (15180 birds) and 12% (7920 birds) of the breeding population each year, respectively, based on the age structure of other species of cormorants (Nelson 2005). Breeding on Siniya Island extends from ca 1 September-15 December (107 days). We considered that breeders were present during the whole breeding period, even when they were not incubating (28 days) and chick-rearing (60 days), due to other gregarious behaviours such as pairing or nest-building.

Assuming they ate mainly anchovy, the total fish consumption of Socotra cormorants from Siniya Island during one breeding season amounted to 5078 tonnes (range: 3506-7263 tonnes), or 47 tonnes per day on average (range: 33-68 tonnes per day).

**Table A1.** Input values (mean ± SD) and references used to calculate time-energy budgets of Socotra cormorants from Siniya Island during the breeding season (BMR: basal metabolic rate; MEI: metabolizable energy intake).

| Parameter | Value | Reference |
| --- | --- | --- |
| Body mass (kg) | 1.52 ± 0.15 | This study |
| Time (h.day^-1^): |  |  |
| - at the colony | 19.6 ± 1.5 | This study |
| - flying | 1.9 ± 0.9 | This study |
| - diving | 0.8 ± 0.3 | This study |
| - on the sea surface | 1.7 ± 0.8 | This study |
| BMR (W.kg^-1^) | 4.8 | Enstipp *et al.* (2005, 2006b) |
| Costs (× BMR) (W.kg^-1^): |  |  |
| - at the colony | 2.0 | Enstipp *et al.* (2006a) |
| - flying | 14.6 | Pennycuick (2008) |
| - diving | 4.2 | Enstipp *et al.* (2006b) |
| - on the sea surface | 2.4 | Enstipp et al. (2006b) |
| Incubation (days)* | 28 | Hockey *et al.* (2005) |
| Early chick-rearing (days)* | 30 | Hockey *et al.* (2005) |
| Late chick-rearing (days)* | 30 | Hockey *et al.* (2005) |
| Fledging (days)* | 60 | Hockey *et al.* (2005) |
| Asymptotic chick mass (g) | 1600 | This study |
| Mean chick MEI (kJ.day^-1^) | 1163 | Visser (2002) |
| Chicks fledged per pair | 1.7 | Muzaffar *et al.* (2012) |
| Anchovy calorific value (kJ.g^-1^ wet mass) | 6.02 ± 0.57 | Balmelli & Wickens 1994 |
| Assimilation efficiency | 0.77 ± 0.34 | Visser (2002) |
| Water temperature (°C) | 27.5 ± 0.9 | This study |
| Fish specific heat capacity (J.g.°C^-1^) | 4.2 | Grémillet *et al.* (2003) |
| Bird body temperature (°C) | 40 | Grémillet *et al.* (2003) |
| Metabolic efficiency | 0.75 | Grémillet *et al.* (2003) |

*Extrapolated from other cormorant species.

**Table A2.** Outputs of the time-energy budget model of Socotra cormorants from Siniya Island during the breeding season (DFI = daily food intake). Maximum or minimum output variation was calculated using a sensitivity analysis approach, by substituting the mean value of model parameters by their mean ± SD (Enstipp et al. 2006b). In order to account for the proportionality of time-activity budget parameters, time at the colony was made to increase or decrease in response to variation in daily time spent flying, diving or on the sea surface. Maximum variation of the model was calculated for the most demanding situation (mean + SD of time-activity budget parameters and body mass; mean ‒ SD of assimilation efficiency and calorific value of prey). The minimum variation of the model was calculated for the least demanding situation (mean ‒ SD of time-activity budget parameters and body mass; mean + SD of assimilation efficiency and calorific value of prey).

| Phase | DFI  (g.day^-1^) | Minimum DFI (g.day^-1^) | Maximum DFI (g.day^-1^) |
| --- | --- | --- | --- |
| Incubation | 429 | 284 | 631 |
| Early chick-rearing | 641 | 470 | 877 |
| Late chick-rearing | 716 | 501 | 1015 |
| Non-breeding | 429 | 284 | 631 |

**References**

Balmelli, W., Wickens, P.A. (1994) Estimates of daily ration for the South African (Cape) fur seal. *South African Journal of Marine Science*, 14,151–157.

Daunt, F., Wanless, S., Harris, M.P., Money, L., Monaghan, P. (2007) Older and wiser: improvements in breeding success are linked to better foraging performance in European shags. *Functional Ecology*, 21, 561–567.

Enstipp, M.R., Grémillet, D., Lorentsen, S.-H. (2005) Energetic costs of diving and thermal status in European shags (*Phalacrocorax aristotelis*). *Journal of Experimental Ecology*, 208, 3451–3461.

Enstipp, M.R., Grémillet, D., Jones, D.R. (2006a) The effects of depth, temperature and food ingestion on the foraging energetics of a diving endotherm, the double-crested cormorant (*Phalacrocorax auritus*). *Journal of Experimental Ecology*, 209, 845–859.

Enstipp, M.R., Daunt, F, Wanless, S., Humphreys, E.M., Hamer, K.C., Benvenuti, S., Grémillet, D. (2006b) Foraging energetics of North Sea birds confronted with fluctuating prey availability. *Top predators in marine ecosystems: their role in monitoring and management.* (eds S. Boyd, S. Wanless & C.J. Camphuysen), pp. 191–210. Cambridge University Press, Cambridge, UK.

Fort, J., Porter, W.P., Grémillet, D. (2011) Energetic modelling: a comparison of the different approaches used in seabirds. *Comparative Biochemistry and Physiology Part A*, 158, 358–365.

Grémillet, D. (1997) Catch per unit effort, foraging efficiency and parental investment in breeding great cormorants (*Phalacrocorax carbo carbo*). *ICES Journal of Marine Science*, 54, 635–644.

Grémillet, D., Wright, G., Lauder, A., Carss, D.N. and Wanless, S. (2003). Modelling the daily food requirements of wintering great cormorants: a bioenergetics tool for wildlife management. *Journal of Applied Ecology*, 40, 266–277.

Hockey, P.A.R., Dean, W.R.J. & Ryan, P.G. (2005) *Roberts birds of southern Africa.* The Trustees of the John Voelcker Bird Book Fund, Cape Town, South Africa.

Muzaffar, S.B., Gubiani, R. & Benjamin, S. (2012) Reproductive performance of the Socotra cormorant (*Phalacrocorax nigrogularis*) on Siniya Island, United Arab Emirates: planted trees increase hatching success. *Waterbirds*, 35, 626–630.

Nelson, J.B. (2005) *Pelicans, cormorants and their relatives: the pelecaniforms*. Oxford University Press, Oxford, UK.

Kato, A., Watanuki, Y., Nishiumi, I., Kuroki, M., Shaughnessy, P., Naito, Y. (2000) Variation in foraging and parental behaviour of king cormorants. *Auk*, 117, 718-730.

Pennycuick, C.J. (2008) *Modelling the flying bird.* Elsevier, San Diego, California, USA.

Visser, G.H. (2002) Chick growth and development in seabirds. *Biology of marine birds* (eds E.A. Schreiber & J. Burger), pp. 439-465. CRC Press, London, UK.
